# Ag^+^-Mediated Folding of Long Polyguanine Strands to Double and Quadruple Helixes

**DOI:** 10.3390/nano14080663

**Published:** 2024-04-11

**Authors:** Liat Katrivas, Anna Makarovsky, Benjamin Kempinski, Antonio Randazzo, Roberto Improta, Dvir Rotem, Danny Porath, Alexander B. Kotlyar

**Affiliations:** 1 Department of Biochemistry and Molecular Biology, George S. Wise Faculty of Life Sciences and Nanotechnology Center, Tel Aviv University, Ramat Aviv, Tel-Aviv 6997801, Israel; liatkatrivas@mail.tau.ac.il (L.K.); kempinski@mail.tau.ac.il (B.K.); 2 Institute of Chemistry and The Center for Nanoscience and Nanotechnology, The Hebrew University of Jerusalem, Jerusalem 7610001, Israel; anna.makarovsky@mail.huji.ac.il (A.M.); rotem.dvir@mail.huji.ac.il (D.R.); 3 Department of Pharmacy, University of Naples Federico II, Via D. Montesano 49, I-80131 Napoli, Italy; antonio.randazzo@unina.it; 4 Istituto di Biostrutture e Bioimmagini-CNR (IBB-CNR), Via De Amicis 95, I-80145 Napoli, Italy; robimp@unina.it

**Keywords:** homoguanine DNA, silver-mediated base pairing, chirality, AFM, STM

## Abstract

Metal-mediated base pairing of DNA has been a topic of extensive research spanning over more than four decades. Precise positioning of a single metal ion by predetermining the DNA sequence, as well as improved conductivity offered by the ions, make these structures interesting candidates in the context of using DNA in nanotechnology. Here, we report the formation and characterization of conjugates of long (kilo bases) homoguanine DNA strands with silver ions. We demonstrate using atomic force microscopy (AFM) and scanning tunneling microscope (STM) that binding of silver ions leads to folding of homoguanine DNA strands in a “hairpin” fashion to yield double-helical, left-handed molecules composed of G-G base pairs each stabilized by a silver ion. Further folding of the DNA–silver conjugate yields linear molecules in which the two halves of the double helix are twisted one against the other in a right-handed fashion. Quantum mechanical calculations on smaller molecular models support the helical twist directions obtained by the high resolution STM analysis. These long guanine-based nanostructures bearing a chain of silver ions have not been synthesized and studied before and are likely to possess conductive properties that will make them attractive candidates for nanoelectronics.

## 1. Introduction

Homoguanine poly(dG) DNA strands are known to self-hybridize in aqueous solutions to form either inter- or intra-molecular structures composed of stacked guanine (G)-tetrads [1,2], G4. Each of the four guanines in the tetrad interacts with the others by means of hydrogen bonds, yielding a stable planar structure. G4-DNAs are stabilized by dipole–ion interactions between the monovalent cations (commonly K^+^ and Na^+^) and G-bases in the central cavity of the DNA [3,4,5]. Long G4-DNAs composed of a large number (hundreds to thousands) of tetrads have been reported [6,7]. These molecules are characterized by great mechanical and thermodynamic stability, e.g., the melting temperature of G4-DNA molecules is much higher than that of DNA duplexes of the same length [8,9,10,11]. Direct electrical measurements on single G4-DNA molecules have demonstrated relatively high electronic conductivity [12,13], which along with the high stability of the molecules, make G4-DNA a candidate for molecular electronics [14].

DNA conductivity can be enhanced by conjugation of the nucleic acid molecules with noble metal particles or metal ions [14,15,16,17]. Silver ions (Ag^+^) are known to tightly and selectively bind to the cytosine and guanine bases composing DNA [18,19]. Stable double-stranded (ds) conjugates between short (tens of bases) homoguanine or homocytosine oligonucleotide and silver ions composed of Ag^+^-mediated homo base pairs, G-Ag^+^-G [20,21] or C-Ag^+^-C [22,23,24,25,26], have been previously reported. These conjugates are thermodynamically more stable than the canonical poly(guanine)-poly(cytosine) duplex [25] and G-quadruplex [20] structures. Most of studies have been conducted on conjugates of relatively short (several nucleotides) G-oligonucleotides with silver ions mainly due to spontaneous folding of long poly (dG)-strands into complex structures: G-guadruplexes and G8-DNA [27].

Here, we report the synthesis and properties of novel conjugates of long (thousands of nucleotides) homoguanine, poly(dG), strands with silver ions. Using circular dichroism (CD) spectroscopy, atomic force microscopy (AFM) and scanning tunneling microscopy (STM), we have demonstrated that in the presence of silver ions, a poly(dG) strand folds on itself in a hairpin fashion, yielding a left-handed double-helical polymer, composed of a large number of guanine base pairs, each stabilized by Ag^+^, G-Ag^+^-G. The two halves of the double-helical polymer can twist right handedly one over the other to form a compact four-stranded structure with two silver ions per cross-section.

## 2. Materials and Methods

### 2.1. Materials

Unless otherwise stated, the reagents were obtained from Sigma-Aldrich (St. Louis, MO, USA) and were used without further purification. Klenow fragment exonuclease minus of DNA polymerase I, *E. coli* lacking the 3′→5′ exonuclease activity (Klenow exo^−^) was purchased from Lucigen Corporation (Middleton, WI, USA). Oligonucleotides were purchased from Integrated DNA Technologies, Inc. (Hayward, CA, USA) unless stated otherwise.

### 2.2. DNA Synthesis

The long poly(dG) strands were prepared from poly(dG)-poly(dC) molecules synthesized as follows: (dG)_12_ and (dC)_12_ oligonucleotides were hybridized and used as primer for synthesis of poly(dG)-poly(dC) as previously described [28]. The assay buffer contained 60 mM Tris-HCl (pH 7.5), 3.2 mM MgCl_2_ and 5 mM dithiothreitol (DTT). For synthesis of 1400 bp poly(dG)-poly(dC), 0.1 µM (dG)_12_-(dC)_12_, 1 mM dGTP, 1 mM dCTP and 0.06 U/µL Klenow exo^−^ were added to the buffer. The polymerase reaction was conducted at 4 °C for 2 h. For synthesis of 100 bp poly(dG)-poly(dC), 2.5 µM (dG)_12_-(dC)_12_, 0.25 mM dGTP, 0.25 mM dCTP and 0.1 U/µL Klenow exo^−^ were added to the assay buffer. The incubation was kept at 4 °C for 5 min and then transferred to 25 °C for another 30 min. The synthesized molecules were chromatographed on a 25 mL Sepharose CL-2B column (1 × 5 cm) equilibrated with 20 mM HEPES-K (pH 7.5). The molecules were collected in the void volume.

### 2.3. Separation of Poly(dG)-poly(dC) Strands

The strands of the synthesized poly(dG)-poly(dC) were separated from each other and purified using anion exchange chromatography on a Tosoh Bioscience LLC (Tokyo, Japan) TSKgel DNA-STAT (0.46 × 10 cm) column using a Thermo Finnigan Surveyor HPLC system (Waltham, MA, USA) equipped with a PDA detector. Peaks were identified by their retention times obtained from the absorbance at 260 nm. The elution was in 0.1 M LiOH and 10% acetonitrile with a linear LiCl gradient from 0 to 2 M at a flow rate of 0.5 mL/min for 120 min.

### 2.4. Preparation of G-Ag^+^-G Conjugates

Poly(dG) strands collected from HPLC (see above) were chromatographed on a prepacked DNA Grade Sephadex G-25 NAP-25 desalting column (Cytiva, Marlborough, MA, USA) equilibrated with 5 mM Tris-NO_3_ (pH 7.5). The absorption spectrum of the void volume fraction containing DNA was measured on a Scinco (Seoul, Republic of Korea) S-3100 spectrophotometer; the concentration of the strands (in G-nucleotides) was calculated using an extinction coefficient of 11.8 mM^−1^ cm^−1^ at 260 nm. AgNO_3_ was added (at various concentrations) to the strand solution and the sample was incubated in a PCR tube for 10 min at 95 °C in a dry bath. The sample was slowly (~5 h) cooled to an ambient temperature of ~25 °C.

### 2.5. CD Spectroscopy

CD spectra were recorded at 25 °C with a Chirascan™ (Applied Photophysics, Surrey, UK) circular dichroism spectrometer using a (1 × 0.4 cm) quartz cuvette. Each spectrum was recorded and was an average of five measurements. Recording specifications were as follows: wavelength step, 1 nm; settling time, 0.333 s; average time, 1.0 s; bandwidth, 1.0 nm; path length, 0.4 cm.

### 2.6. Atomic Force Microscopy (AFM)

DNA samples (10–20 μL) in 2 mM Tris-NO_3_ (pH 7.5) were diluted into 100 μL of 2 mM Mg(NO_3_)_2_ and deposited on freshly cleaved mica for 5 min. The surface was then washed with distilled water and dried with N_2_ flow. AFM imaging was performed on a Solver PRO AFM system (NT-MDT, Zelenograd, Russia), in a semi-contact mode, using 130 μm-long Si-gold-coated cantilevers (ScanSens, Munich, Germany) with a resonance frequency of 70–180 kHz. The images were “flattened” (each line of the image was fitted to a second-order polynomial, and the polynomial was then subtracted from the image line) with Nova image processing software (Version 3.5, NT-MDT, Russia) and analyzed using Nanotec Electronica S.L (Madrid, Spain) WSxM 4.0 Beta 9.3 imaging software [29].

### 2.7. Scanning Tunneling Microscopy

STM imaging was performed with Scienta Omicron (Uppsala, Sewden) LT-UHV-STM on molecules adsorbed on a gold substrate. Prior to deposition, a clean gold surface was flame annealed to form large flat grains. The substrate was then treated with 1 mM cystamine for 30 min. A solution of G-rings (30 µL; 20 mOD at 260 nm) in 3 mM KNO_3_ was incubated on the substrate for 8 min, rinsed with distilled water and dried with N_2_ gas. All the measurements were conducted at 25 °C under high vacuum conditions (1–6 × 10^−8^ mbar). Optimal imaging was obtained with a standard Omicron tungsten tip at a bias voltage of 2.5–2.8 V and set current of 50–70 pA. The height and helical pitch distance were calculated by profiling the molecule across or along its axis, respectively.

### 2.8. Computational Details

The large size of the systems under study (the minimal meaningful models include at least ~100 atoms) makes QM calculations rather challenging and restricts the possible choice for the electronic methods to be used. We have thus resorted to the cost-effective density functional theory (DFT) calculations for geometry optimizations and to time-dependent DFT (TD-DFT) ones to compute the absorption and ECD spectra, with the M052X functional [30] and two different basis sets/pseudopotential labeled as ‘GEN’ (def2-SVP for C, N, O, H, P, Na, Ag + ECP def2-SVP for Ag) and ‘GEN1′ (6–31+G(d,p) for C, N, O, H, P, Na and LANL2DZ + ECP LANL2DZ for Ag). Solvent effects have been included using the PCM model [31]. In order to simulate the spectrum each transition was broadened with a Gaussian with half width at half maximum (HWHM) of 0.2. In addition to the possible limitations of the computational methods adopted, the lack of vibrational and thermal effects in our calculations led to a systematic blue shift of the computed vertical transition energies with respect to the maximum of the absorption band [32]. As a consequence, the computed spectra (as expected) were blue-shifted with respect to the experimental ones.

## 3. Results and Discussion

**Figure 1:**
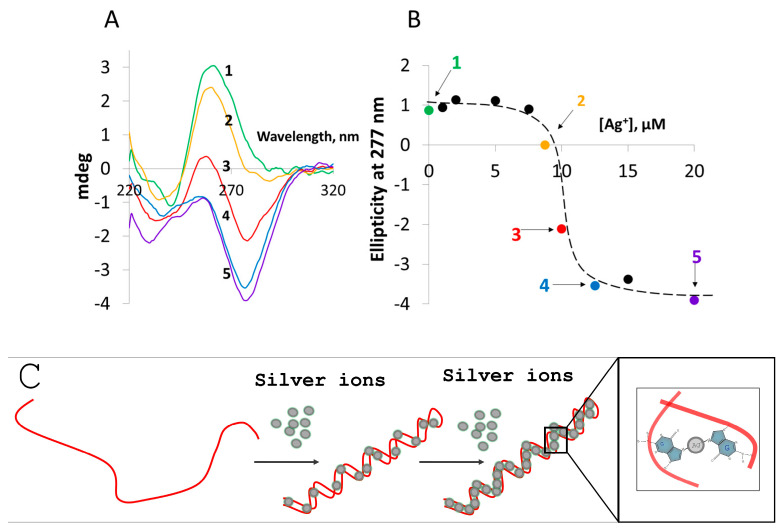
(**A**) CD spectra of a 100-base G-strand conjugated with Ag^+^ at different G-nucleoside to Ag^+^ ratios. A 20 µM (in G-nucleosides) G-strand solution (spectrum 1) was incubated for 10 min at 95 °C with: 1, 2, 5, 7.5, 8.75, 10, 12.5, 15 and 20 µM AgNO_3_ followed by slow (5–6 h) cooling of the sample to ambient temperature. Spectra 2, 3, 4 and 5 correspond to 8.75, 10, 12.5 and 20 µM AgNO_3_, respectively. (**B**) Dependence of the CD signal amplitude at 277 nm on Ag^+^ concentration in the incubation. (**C**) Schematic representation of the silver ions interaction with G strand with the increase in silver ion concentration. The right part of the panel shows an enlarged area of the silver-mediated interaction of two G bases in the hairpin.

**Figure 2:**
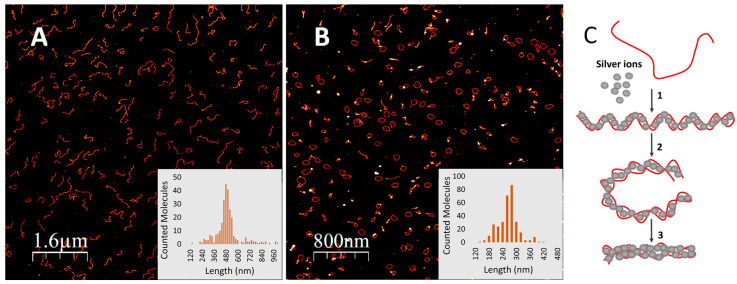
Folding of a poly(dG)-Ag^+^ conjugate. (**A**) AFM image of ~1400-base pair poly(dG)-poly(dC) molecules. Inset: contour length histogram of the molecules; the average length of 278 individual molecules is 490 ± 131 nm. (**B**) AFM image of the poly(dG)-Ag^+^ conjugate prepared from the above poly(dG)-poly(dC) molecules (**A**) as described in Materials and Methods. The G-strand was prepared from 1400-base pair poly(dG)-poly(dC) using ion-exchange HPLC under alkaline conditions and then mixed with Ag^+^ as described in Section 2 (see Supporting Information) and Figure S1. Inset: Contour length histogram of the molecules; the average length of 316 individual molecules is 255 ± 43 nm. (**C**) Scheme depicting conjugation of a G-strand (red curve) with silver ions (grey spheres), folding of the conjugate into a circular ring-shaped structure, G-ring (reaction 2) and subsequent folding of the ring into a supercoiled compact helix (reaction 3).

**Figure 3:**
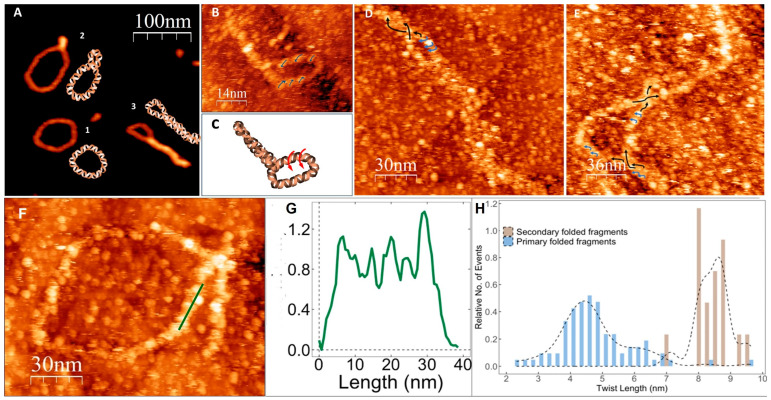
Conjugate analysis using AFM (**A**) and high-resolution STM (**B**,**D**–**F**) images and schemes of individual G-Ag^+^-G molecules at different stages of the folding process. (**A**) Circular (1), slightly twisted (2) and almost fully twisted (3) rod-like molecules along with corresponding schematic images. (**C**) Schematic drawing of the molecule seen in (**B**). (**B**,**D**,**E**) High-resolution STM scans of the primary (blue arrows) as the secondary (black arrows) folded regions in poly(G-Ag^+^-G) molecules. Scans were analyzed for the visual assessment of their twist directionality for left- or right-hand twist. A total of 12 out of 12 primary folded regions analyzed were left-handed, and 20 out of 20 secondary folded regions were right-handed. (**F**) STM image of ring-shaped primary folded molecule. (**G**) Height profile taken along the green line seen in (**F**). (**H**) Twist length distribution histogram for primary and secondary folded areas in poly(G-Ag^+^-G) conjugate. About 90 primary (ring-shaped) folded fragments areas in 22 individual molecules were analyzed. The twist periodicity of 4.8 ± 0.7 nm was estimated by analysis described in (**B**)). The twist periodicity of 8.5 ± 0.4 nm (see brown bars) was estimated for the secondary folded (rod-shaped) fragments using similar analysis of ≈20 areas in 16 individual molecules.

Incubation of long (hundreds of nucleotides) poly(dG) with silver ions, conducted as described in Materials and Methods (see Supporting Information), led to significant changes in the circular dichroism (CD) spectrum of the DNA (Figure 1A). The negative ellipticity (which is a measure of the difference in absorbance of left-handed circularly polarized light compared to right-handed circularly polarized light) at 277 nm increased progressively with the ratio of silver ions to guanine (G) bases in the 0 to 0.5 range. Significant changes observed in the CD spectrum suggest that binding of silver ions considerably affects the DNA’s secondary structure. Further increase in the ratio beyond 0.5 did not lead to any change in either the shape of the CD spectrum or the amplitude of the signal at 277 nm (Figure 1B). Binding of silver ions lead to folding of the G-strand into ring-shaped structures as evident from AFM scans (Figure 2B); the average contour length of the rings is 255 ± 43 nm (Figure 2B, inset). This value corresponds to about half the length of the parent 1400 bp poly(dG)-poly(dC) molecule, 490 ± 131 nm (Figure 2A, inset) used for preparation of the homoguanidine strand. This suggests that in the conjugate the G-strand is folded on itself such that the two halves, oriented in opposite directions, are hybridized through Ag^+^-mediated G-G pairing to form a helix (Figure 2C, reaction 1). The edges of the G-Ag^+^-G molecules approach each other, probably due to mechanical tension caused by the helical twist, resulting in formation of a ring-shaped structure (Figure 2C, reaction 2). The height of the structure is identical to that of canonical ds DNA [33] measured by AFM (Figure S2), supporting the ds nature of the ring-shaped molecules. The circular structures can then further fold into thicker, linear rod-like structures (Figure 2C, reaction 3) seen in Figure 3. Different intermediate stages of the folding process are seen in Figure 3A. The length of the super-coiled structures is 109 ± 28 nm (Figure S3), which is equal to about one fourth the length of the parent poly(dG)-poly(dC) molecules (Figure 2A).

The apparent height of the hairpin structures, 1.57 ± 0.28 nm (Figure S2), is larger than that of ds DNA (Figure S4) and the ring-shaped structures (Figure 2B). In order to determine the structure of the folded molecules, we used high-resolution STM. The resolution achieved by STM enabled us to resolve the periodic structure within individual molecules, reminiscent of the molecule’s helix, in the hairpin ds ring-shaped G-Ag^+^-G area of the molecules (Figure 3B) and to estimate the twist lengths of the helix as well as the direction of their helical twist (Figure 3). Analysis of the images revealed that the G-Ag^+^-G double helix twists in a counterclockwise (left-handed) direction (Figure 3B,D,E). This is in good agreement with the results of the CD spectroscopy, showing a strong negative signal of G-Ag^+^-G molecules in the 250–300 nm range of the spectrum (Figure 1A). DFT and time-dependent DFT calculations (see Figures S5–S9 for details) applied to short models of dG-oligonucleotides (containing up to 8 bases) also support that the dG-strands are associated into duplexes characterized by a counterclockwise direction of the helical twist (see Supporting Information). The results of the calculations also indicate side-to-side interaction between the duplex molecules mediated by Ag^+^-ions. These interactions can stabilize the rod-like structures formed during folding of the ring-shaped ones (Figure 2C, reaction 3). The high resolution STM analysis of the folded ring-shaped G-Ag^+^-G structures revealed the presence of periodic peaks along the contour of the molecule. Taking a height-profile line along the molecule, one may extract the distance between these peaks and determine the twist length of the helix.

This analysis, performed on over 50 individual ring-shaped molecules, yielded an average twist length of 4.8 ± 0.7 nm (Figure 3). The measured value appears to be somewhat larger than that of canonical ds DNA (~3.4 nm). The larger twist length of the G-Ag^+^-G structure was also predicted by the DFT calculations (see Supporting Information). Similar analysis of about 40 rod-shaped structures that resulted from folding of G-rings into super-coiled compact structures as well as rod-shaped fragments of the partly folded molecules, such as the one shown in the bottom-right corner of Figure 3A, revealed a twist length of 8.5 ± 0.7 nm for the secondary folding. The two double-helical fragments of the molecule are coiled together like the strands in a rope forming a coiled coil rod-like structure. The analysis of STM scans of the twist length of the hairpin double helix and the coil coiled structures is presented in Figure 3. STM scans of 32 molecules and areas in molecules obtained at high resolution were analyzed for their directionality (see example in Figure 3B and scheme in Figure 3C). A total of 12 out of 12 molecular areas showed a left-handed twist of the primary hairpin supercoiling and 20 out of 20 rod supercoiled molecules exhibited right-handed secondary coiling (Figure 3D,E as examples).

We have demonstrated earlier that poly(dG)-based structures are characterized by higher charge conductivity than ds DNA [13,14]. The novel stable conjugates of homoguanine polymer with redox-active silver cations demonstrated here are expected to possess improved electrical properties compared to previously reported G4-DNA molecules [13,14], making them attractive candidates for molecular nanoelectronics.

## 4. Conclusions

In summary, this study reports the folding of very long (thousands of bases) homoguanine strands, induced by silver ions. As evident from CD measurements and AFM imaging analysis, the G-strand folds on itself, leading to the formation of a double-helical structure composed of G-G base pairs, each stabilized by an Ag^+^-ion. The opposite ends of the molecule are approaching each other, leading to the formation of an opened ring-shaped structure. Further twisting of this circular structure in a screw-like fashion resulted in compact and stiff rod-shaped molecules; each cross-section of the completely folded molecule contains four G bases and two red/ox active silver ions. The poly(dG)-Ag^+^-based conjugates are stiff and very stable under ambient and elevated temperatures (90 °C). High-resolution STM supported by DFT modeling revealed that the molecules adopt a counter-clockwise, left-handed helical conformation in their primary folding and a right-hand directionality in the secondary folding. These new conjugates are likely to be more conductive than previously reported DNA-based molecules and be used as elements in nanobioelectronic devices and nanobiosensors.

## Data Availability

Data are contained within the article and supplementary materials.

## Supplementary Materials

The following supporting information can be downloaded at: https://www.mdpi.com/article/10.3390/nano14080663/s1, Figure S1. Anion-exchange chromatography of 1400 bp poly(dG)-poly(dC). The separation was conducted in 0.1 M LiOH and 10% acetonitrile. The strands were eluted from ion-exchange DNA-STAT (TOSOH, Japan) column at ambient temperature with a linear LiCl gradient from 0 to 2 M for 120 min at a flow rate of 0.5 mL/min. The elution was monitored at 260 nm using Thermo Finnigan Surveyor HPLC system equipped with PDA detector. Figure S2. Height distribution of ring-shaped G-Ag^+^-G molecules. The heights were obtained from AFM scans of the molecules shown in Figure 2B. The average height of the molecules was calculated using WSxM software2. The calculated average height is 0.68 ± 0.11 nm (n = 68). Figure S3. Length distribution of rod-shaped supercoiled G-Ag^+^-G structures. The length values were obtained from analysis of AFM scans similar to that shown in Figure 2B. The contour length of completely folded rod-shaped molecules seen on the surface (see Figure 2B) together with the ring-shaped ones was measured using WSxM software2. The average length of the molecules is 109 ± 28 nm (n = 62). Figure S4. Height distribution of rod-shaped supercoiled G-Ag^+^-G structures. The height values were obtained from analysis of AFM scans similar to that shown in Figure 2B. The average height of completely folded rod-shaped molecules seen on the surface (see Figure 2B) together with the ring-shaped ones was measured using WSxM software2. The calculated average height is 1.57 ± 0.28 nm (n = 55). Figure S5. Schematic presentation of 2GAg^+^G (upper panel) and 4GAg^+^G (lower panel) in minimum energy configurations. Color codes for the atoms are as follows: carbon (green), oxygen (red), nitrogen (cyan), hydrogen (white), phosphate (gold), silver (yellow). Hydrogen atoms are omitted, and hydrogen bonds are marked with red dashed lines. Coordination interactions of the silver ion are depicted in purple (see text for details). Figure S6. Schematic presentation of 3GAg^+^G-met (upper panel) and 4GAg^+^G-met (lower panel) in minimum energy configurations. Left-handed double-stranded Z-DNA was used as starting geometry for the optimizations. Color codes for the atoms are as follows: carbon (green), oxygen (red), nitrogen (cyan), hydrogen (white), phosphate (gold), silver (yellow). Hydrogen atoms are omitted, and hydrogen bonds are marked with red dashed lines. Coordination interactions of the silver ion are depicted in purple (see text for details). Terminal CH2-OCH3 groups are marked by blue dotted circles. Figure S7. Computed ECD spectra for 2G-Ag^+^-G, 3G-Ag^+^-G, and 4G-Ag^+^-G species ∆ε in 10^−40^ esu^2^ cm^2^. PCM/TD-M052X calculations with the GEN basis set (see text for details). Each stick transitions broadened with a Gaussian with half-width half-maximum (HWHM) of 0.2 eV. Figure S8. Computed ECD spectra for 3G-Ag^+^-G-met and 4G-Ag^+^-G-met species. ∆ε in 10^−40^ esu^2^ cm^2^. PCM/TD-M052X calculations with the GEN basis set (see text for details). Each stick transitions broadened with a Gaussian with HWHM of 0.2 eV. Figure S9. Computed ECD spectra for 3G-Ag^+^-G-met and 3G-Ag^+^-G using different basis sets for geometry optimizations and/or CD calculations. ∆ε in 10^−40^ esu^2^ cm^2^. If not otherwise specified, PCM/TD-M052X calculations were conducted with the GEN basis set (see text for details). Each stick transitions broadened with a Gaussian with HWHM of 0.2 eV. Ref [34] is cited in Supplementary Materials.
