# Supplementary material for: Ag+-Mediated Folding of Long Polyguanine Strands to Double and Quadruple Helixes
Source: Nanomaterials (Basel). 2024 Apr 11;14(8):663. doi: 10.3390/nano14080663 (PMC11055002; doi:10.3390/nano14080663)
Supplement: Supplementary file 1 [file nanomaterials-14-00663-s001.zip › nanomaterials-2944611-supplementary.pdf]

Supporting Information

Ag+-Mediated Folding of Long Polyguanine Strands to Double and Quadruple Helixes

Liat Katrivas [1], Anna Makarovsky [2], Benjamin Kempinski [1], Antonio Randazzo [3], Roberto Improta [4], Dvir Rotem [2], Danny Porath [2,*] and Alexander B. Kotlyar [1,*]

Figures S1-S6

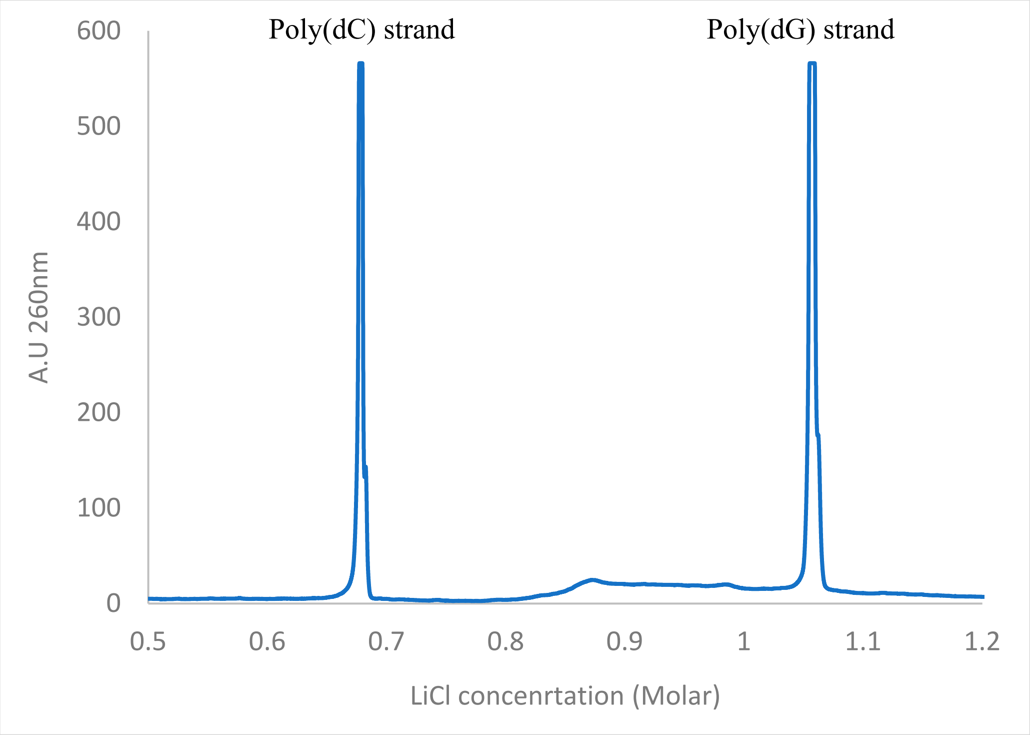

Figure S1. Anion-exchange chromatography of 1400 bp poly(dG)-poly(dC). The separation was conducted in 0.1M LiOH and 10% acetonitrile. The strands were eluted from an ion-exchange DNA-STAT (TOSOH, Japan) column at ambient temperature with a linear LiCl gradient from 0 to 2M for 120 minutes at a flow rate of 0.5 mL/min. The elution was monitored at 260 nm using a Thermo Finnigan Surveyor HPLC system equipped with a PDA detector.

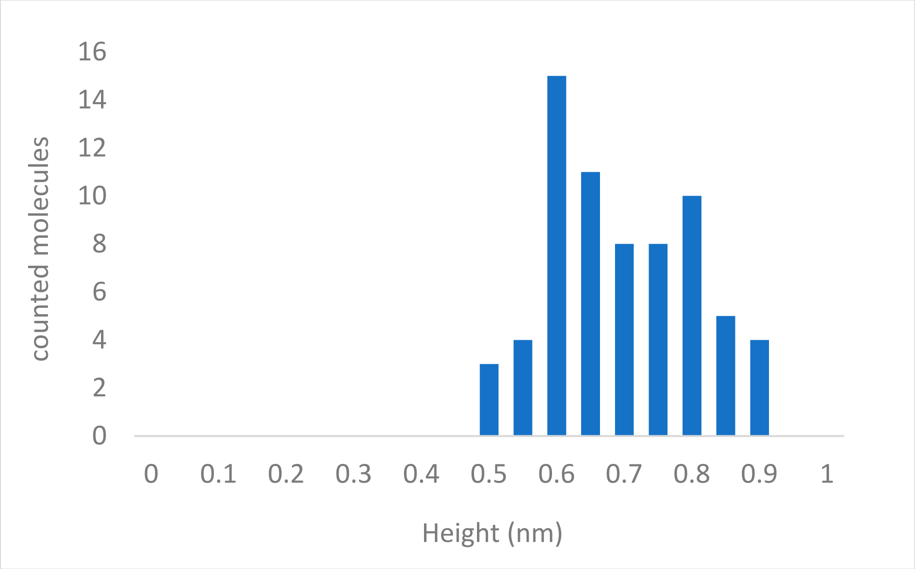

Figure S2. Height distribution of ring-shaped G-Ag+-G molecules. The heights were obtained from AFM scans of the molecules shown in Figure 2B. The average height of the molecules was calculated using WSxM software2. The calculated average height is 0.68 ± 0.11 nm (n = 68).

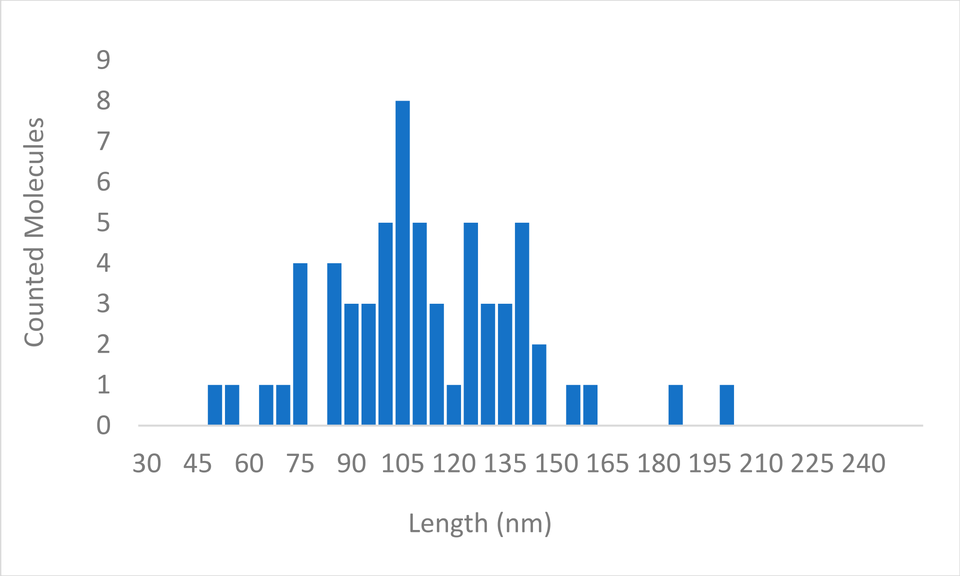

Figure S3. Length distribution of rod-shaped supercoiled G-Ag+-G structures. The length values were obtained from analysis of AFM scans similar to that shown in Figure 2B. The contour length of completely folded rod-shaped molecules seen on the surface (see Figure 2B) together with the ring-shaped ones was measured using WSxM software2. The average length of the molecules is 109 ± 28 nm (n = 62).

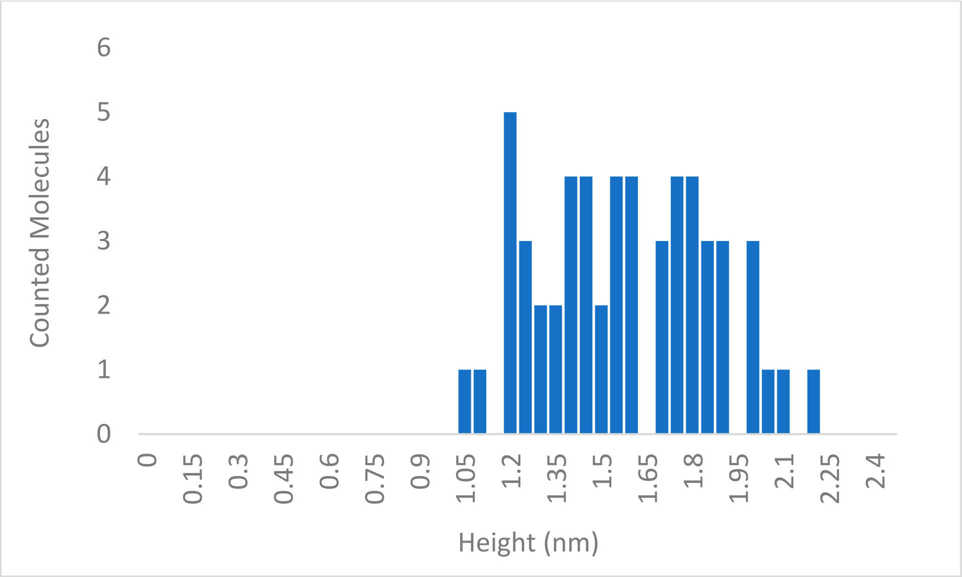

Figure S4. Height distribution of rod-shaped supercoiled G-Ag+-G structures. The height values were obtained from analysis of AFM scans similar to that shown in Figure 2B. The average height of completely folded rod-shaped molecules seen on the surface (see Figure 2B) together with the ring-shaped ones was measured using WSxM software2. The calculated average height is 1.57± 0.28 nm (n = 55).

Computational results (Figures S5-S9)

The models used for the geometry optimizations (illustrated in Figure S7) include two (dG)n strands (n=2,3,4), conjugated with two, three and four Ag+ ions, respectively: 2GAg+G, 3GAg+G and 4GAg+G, respectively. We have also performed similar calculations on methylated 3G and 4G strands (containing O-meth groups at 5′ and 3′ ends of the strand), 3GAg+G-met and 4GAg+G-met). For the starting geometry, we used the left-handed Z-DNA conformation.

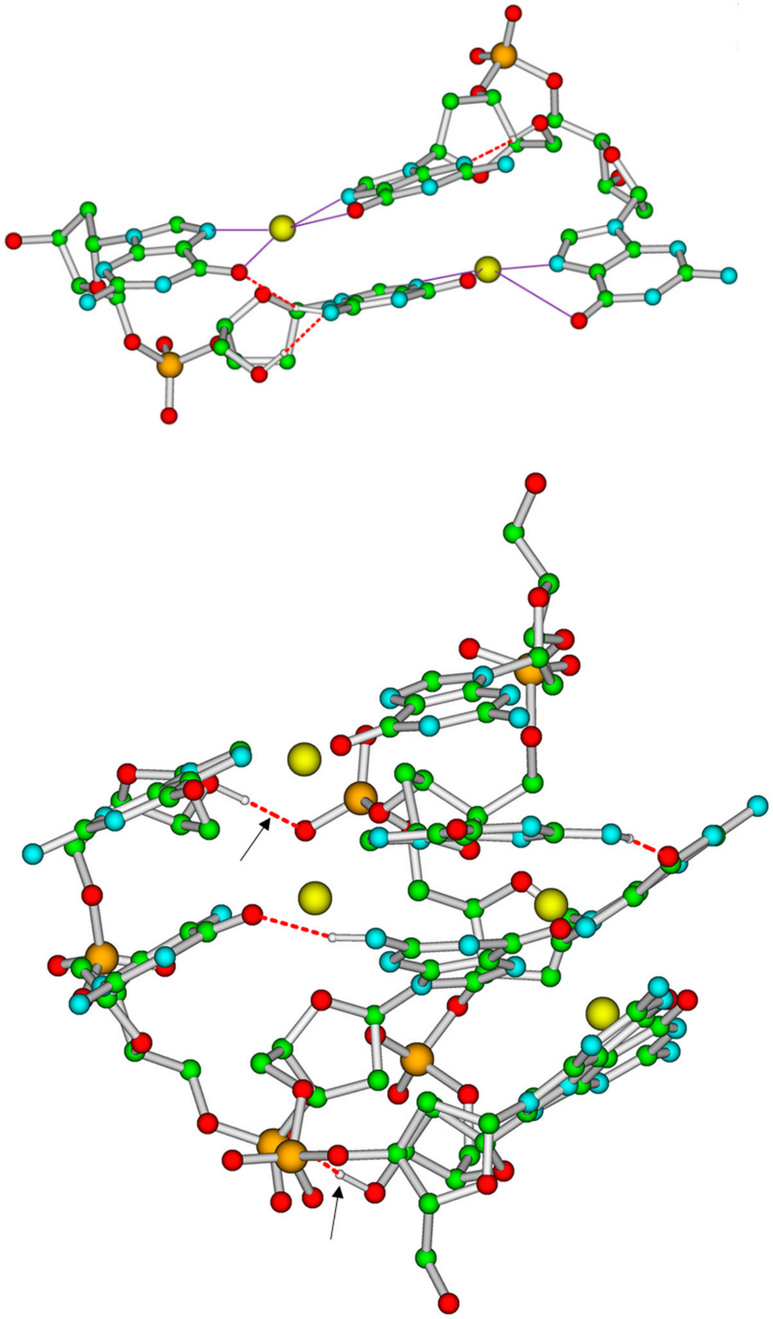

Figure S5. Schematic presentation of 2GAg+G (upper panel) and 4GAg+G (lower panel) in minimum energy configurations. Color codes for the atoms are as follows: carbon (green), oxygen (red), nitrogen (cyan), hydrogen (white), phosphate (gold), silver (yellow). Hydrogen atoms are omitted and hydrogen bonds are marked with red dashed lines. Coordination interactions of the silver ion are depicted in purple (see text for details).

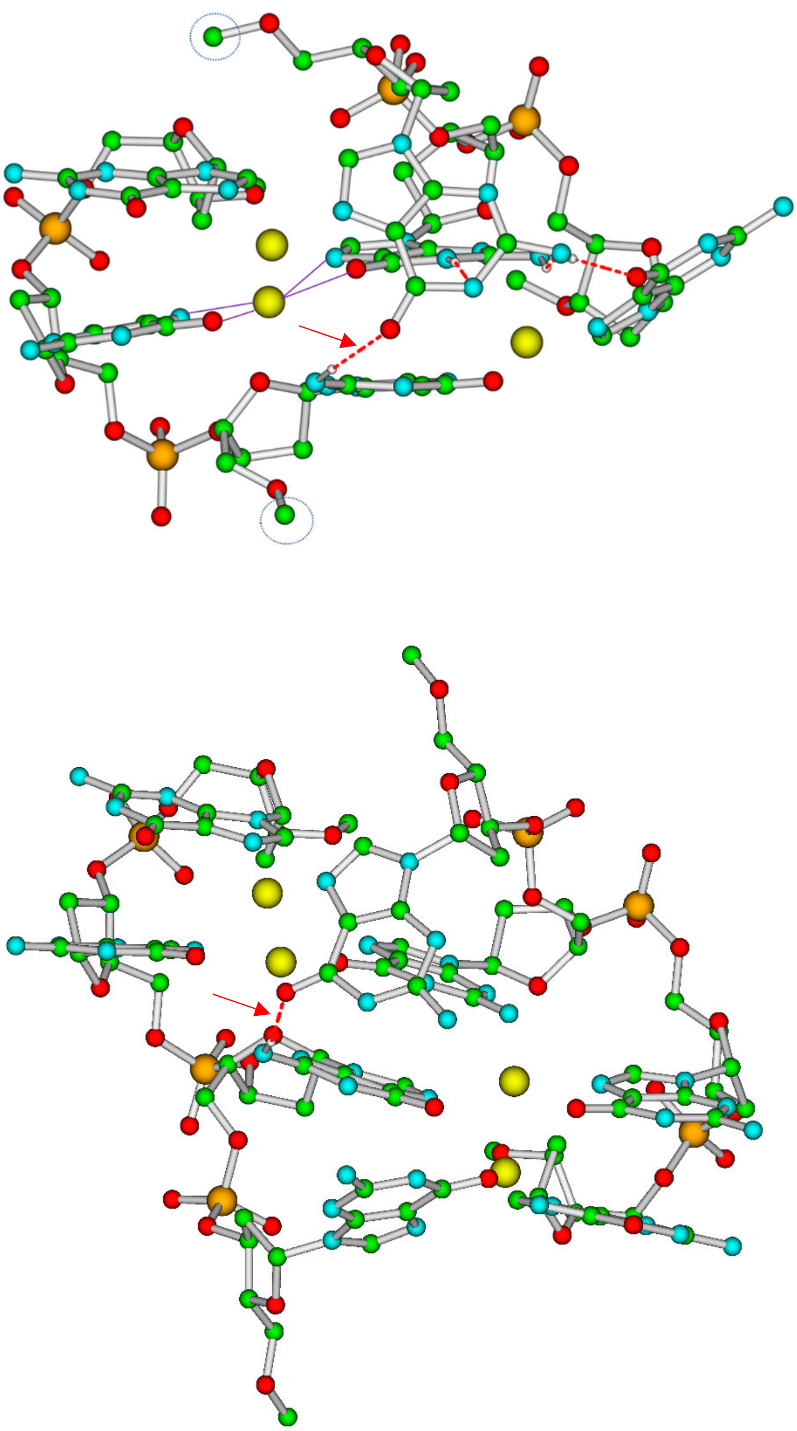

Figure S6. Schematic presentation of 3GAg+G-met (upper panel) and 4GAg+G-met (lower panel) in minimum energy configurations. Left-handed double-stranded Z-DNA was used as starting geometry for the optimizations. Color codes for the atoms are as follows: carbon (green), oxygen (red), nitrogen (cyan), hydrogen (white), phosphate (gold), silver (yellow). Hydrogen atoms are omitted and hydrogen bonds are marked with red dashed lines. Coordination interactions of the silver ion are depicted in purple (see text for details). Terminal CH2-OCH3 groups are marked by blue dotted circles.

In the minimum energy geometry, the two G-strands composing structures depicted in Figures S7 and S8 are running anti-parallel and the carbonyl oxygen and N7 nitrogen of each of two G bases composing the pair are involved in the coordination with the silver ion (shown in purple in Figure S7). This agrees with the X-ray structures available on Ag+-oligonucleotides conjugates. The atoms of the adjacent base pairs can also coordinate the silver ion. In each strand composing the double-stranded (ds) structure there is alternation between syn- and anti-nucleobases. The strands in the structure are antiparallel supporting folding of a long G-strand in a hairpin fashion (Figure 2C). We have included the bulk solvent effects in the calculations by employing the PCM model. However, since we did not account for interactions of individual water molecules with the DNA nucleotides, geometry optimizations converged to minima that correspond to multiple intra-molecular hydrogen bonds, for example, the bond between the terminal OH and/or CH2-OH groups and the oxygen atoms of the phosphate groups (pointed out by arrows in Figure S7). In order to avoid the contribution of these hydrogen bonds (whose role, in any case, is negligible for formation of nanowires) in the folding process, we have also studied the methylated strands, 3G-Ag+-G-met and 4G-Ag+-G-met, in which the terminal groups cannot be involved in formation of the above strong hydrogen bonds. 3G-Ag+-+G-met and 4G-Ag+-+G-met molecules can still form inter-base hydrogen bonds (shown by red arrows in Figure S8), interfering with coordination of the bases with silver ions. Contribution of these interactions into the folding process is, however, rather minor. The estimated average distance between the terminal 5′-and 3′-OCH3 in 4GAg+G-met is ~15.8 Å, corresponding to a pitch of ~4 Å. This result fits nicely with the pitch length estimated by high-resolution STM analysis of long G-Ag+-G molecules (Figure S5C).

Figures S7-S9 show ECD spectra computed for the above models. Our main focus was on the spectral shape at wavelengths above 240 nm since the reliability of our predictions at shorter wavelengths is limited by the number of excited states considered in the calculations. In the presence of silver ions, a negative band appears at wavelengths below 280 nm in the ECD spectrum of the G-strands, which is consistent with the experimental observation (Figure 1A). However, as discussed earlier, the use of M052X functionals [34] and the neglect of vibrational contributions [33] are expected to yield spectra uniformly blue-shifted with respect to the experimental ones. The negative band of the computed ECD spectra of all the models is indeed slightly blue-shifted with respect to the corresponding experimental one. The model exhibiting the worse agreement with the experimental spectral shape is 4G-Ag+-G, for which the negative band is very weak. This inaccuracy is likely due to the distortion of the regular stacking arrangement exhibited by the minimum energy geometry for 4G-Ag+-G. This distortion reflects a tendency of maximizing the effect of 'spurious' intramolecular hydrogen bonds (including those formed by the terminal OH groups) on the results of the calculations. In fact, the spectrum computed for 4G-Ag+-G-met (shown in Figure S8) fits the experimental one (curve 5 in Figure 1) much better than that computed for 4G-Ag+-G. Finally, we have demonstrated that the computed spectral shape is independent of the basis set used in the geometry optimizations and/or in the ECD calculations confirming that our predictions are solid with respect to the size of the basis set. Interestingly, the spectra computed by using the larger GEN1 basis set are closer to the experimental ones (Figure S9), with the 'characteristic' negative peak falling at ~270 nm, independently of the basis set adopted in the geometry optimizations.

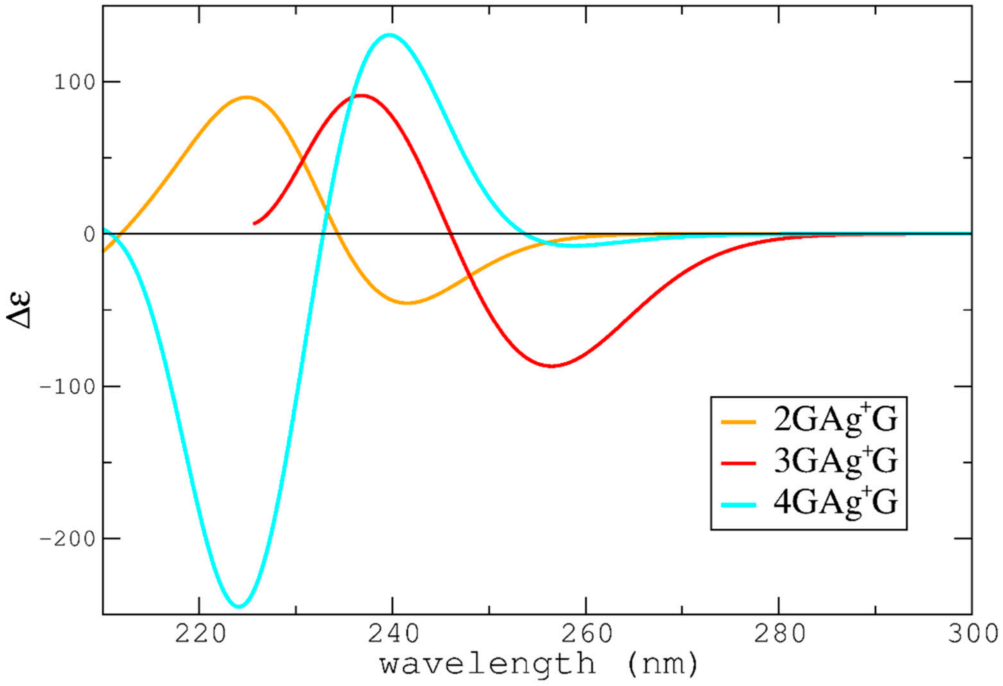

Figure S7. Computed ECD spectra for 2G-Ag+-G, 3G-Ag+-G and 4G-Ag+-G species Δε in 10^{-40} esu^2 cm^2. PCM/TD-M052X calculations with the GEN basis set (see text for details). Each stick transitions broadened with a Gaussian with half-width half-maximum (HWHM) of 0.2 eV.

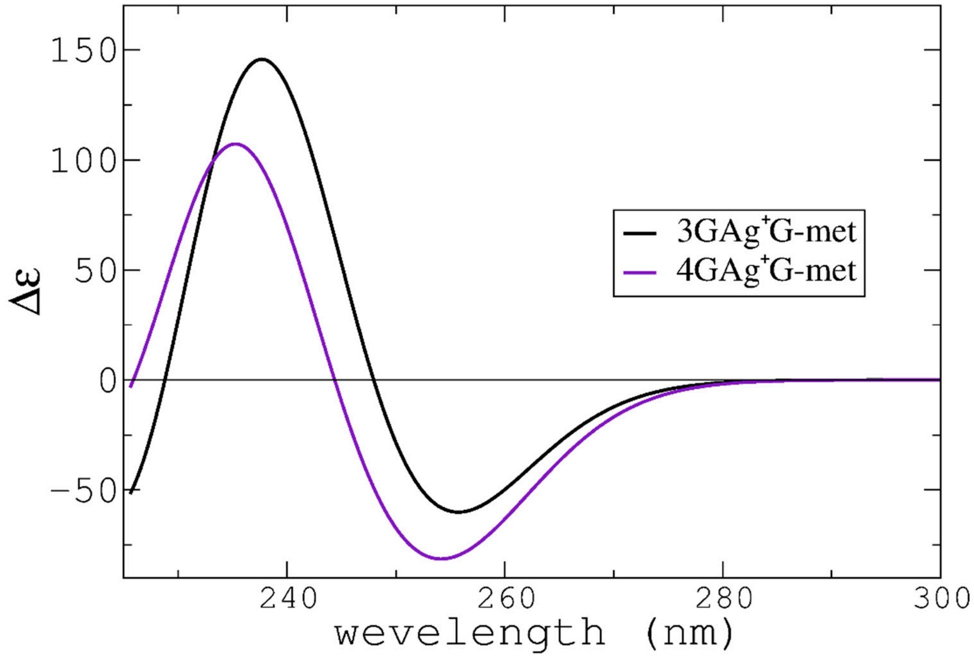

Figure S8. Computed ECD spectra for 3G-Ag+-G-met and 4G-Ag+-G-met species. Δε in 10^{-40} esu^2 cm^2. PCM/TD-M052X calculations with the GEN basis set (see text for details). Each stick transitions broadened with a Gaussian with HWHM of 0.2 eV.

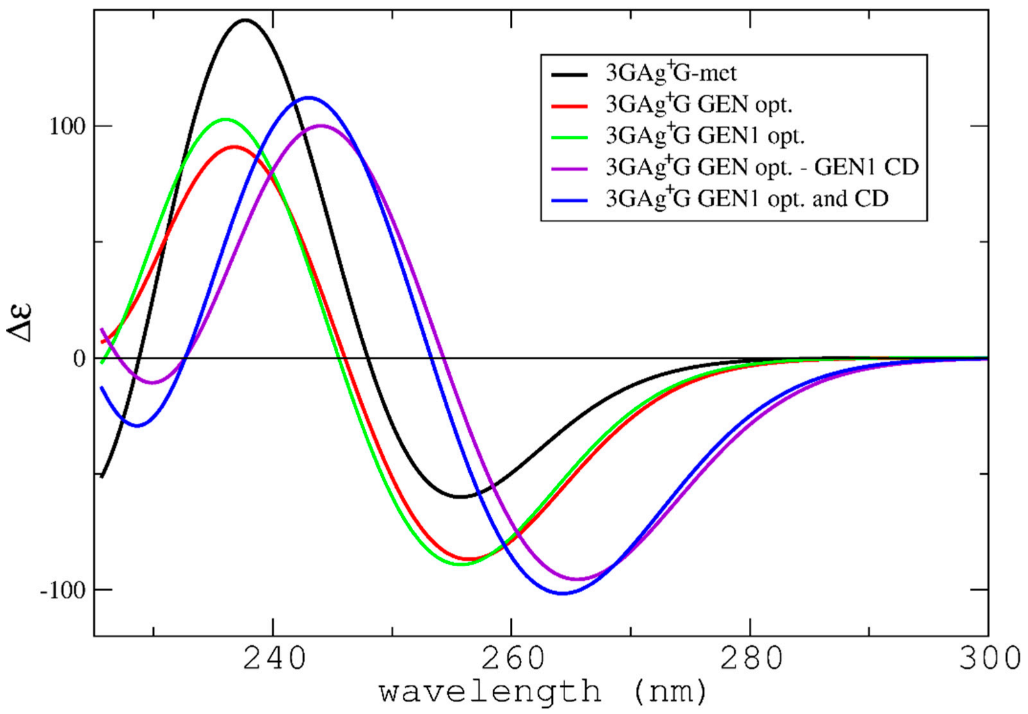

Figure S9. Computed ECD spectra for 3G-Ag+-G-met and 3G-Ag+-G using different basis sets for geometry optimizations and/or CD calculations. Δε in 10^{-40} esu^2 cm^2. If not otherwise specified, PCM/TD-M052X calculations were conducted with the GEN basis set (see text for details). Each stick transitions broadened with a Gaussian with HWHM of 0.2 eV.
